# Case of Poisoning by Arsenic

**Published:** 1849-03

**Authors:** Walter Clegg


*Case of Poisoning by Arsenic, in which the symptoms were unusually delayed.* By WALTER CLEGG, Esq.—The London Lancet for November contains the following remarkable case:

On Sunday, August 27th, at 5 o'clock in the afternoon, a woman requested me to visit her niece, who had, about noon, taken a teaspoonful of white arsenic. I attended, and found a heavy, stupid looking girl sitting in her chair, more asleep than awake. On rousing her, she reeled about the room as if intoxicated; indeed, I suspected poisoning by some narcotic. But she acknowledged having swallowed "white mercury," and a paper packet was brought to me, from which she had taken the poison, containing about ten grains of a white powder. By means of my pocket-lens, I immediately recognized this powder to be arsenious acid. She vomited once after dinner, but there were no further symptoms until half an hour before she died—that is, at noon the following day.

She had no sickness, no pain, no acrid eructations, no burning taste in the mouth; her face was very pale, and she was faint and giddy. The sulphate of zinc, with mucilaginous drinks, soon produced profuse vomiting, and this was kept up for half an hour. Then another medical man arrived with a jar of the hydrated peroxide of iron; and having some pressing professional engagements, I left her with my friend, who administered large doses of the antidote.

At 9 o'clock at night we visited her together. She had experienced no pain; no unpleasant symptom whatever; she was disposed to sleep quietly. At 10 o'clock the next morning the patient's aunt came to say that her niece was quite well, "might she go a gleaning?" Up to half past 11 o'clock she continued more than ordinarily cheerful, and was busied in preparing the family dinner. At half past 11 o'clock she suddenly complained of an excruciating pain in the body, with excessive prostration of strength. She went to her bed room to lie down, and at 12 o'clock was found dead, kneeling by the bedside. Thus she died in about eighteen hours after taking the poison, and within half an hour after the first decided symptom of poisoning was manifested.

*Autopsy, forty-eight hours afterwards.*—The stomach contained half a pint of a thin, dirty green fluid; the mucous coat was much corrugated, having a fungoid appearnce, very soft, and so fragile that a touch of the finger tore it away. Three or four large reddish brown patches were observed, and these extended into the intestine considerably beyond the duodenum. The peritoneal coats of the stomach and bowels were not inflamed; the lungs and the heart were healthy; the head was not inspected.

It only remains for me to add that the matter vomited and the fluids in the stomach and intestines contained abundant evidence of the presence of arsenic. Minute fragments of a white powder were seen adhering to the mucous coat of the stomach, and these, by the ordinary tests, were proved most satisfactorily to be arsenious acid.

*Explanation.*—The lower classes in Lincolnshire are very much addicted to the use of opium and laudanum. I have known women with three shillings a week from the parish, spend half a crown in the purchase of opium. I have reason to believe that this girl was an opium-eater. Did one poison mask the evidences of the other? Did the opium suppress the horrible agonies of arsenical poisoning, thus modifying the symptoms, whilst it had no power to interrupt the effects of the deadly drug?

